# Mental health-related presentations to Australian hospital emergency departments by children and young people, before and after the onset of the COVID-19 pandemic: a systematic review and meta-analysis

**DOI:** 10.1186/s12873-026-01700-x

**Published:** 2026-07-22

**Authors:** Jackson Newberry-Dupé, Simon Craig, Glenn Melvin, Kylie King, Yuxi Li, Alexander Campbell, Tom Shore, Timothy Curtis, Rohan Borschmann

**Affiliations:** 1 Melbourne School of Population and Global Health, The University of Melbourne, Parkville, VIC Australia; 2 Centre for Adolescent Health, Murdoch Children’s Research Institute, Parkville, VIC Australia; 3 Department of Paediatrics, School of Clinical Sciences, Monash University, Clayton, VIC Australia; 4 Paediatric Emergency Department, Emergency Service, Monash Medical Centre, Monash Health, Clayton, VIC Australia; 5 Emergency Research Group, Murdoch Children’s Research Institute, Parkville, VIC Australia; 6 School of Psychology, Deakin University, Burwood, VIC Australia; 7 School of Psychological Sciences, Monash University, Clayton, VIC Australia; 8 Stretton Health Equity, School of Public Health, Adelaide University, Adelaide, SA Australia; 9 Port Macquarie Base Hospital, Mid North Coast Health, Port Macquarie, NSW Australia; 10 Health Service and Population Research Department, Institute of Psychiatry, Psychology & Neuroscience, King’s College London, London, UK; 11 Better Health & Care Hub, King’s College London, London, UK; 12 Department of Psychiatry, University of Oxford, Oxford, UK

**Keywords:** COVID-19, Psychiatry, Mental health, Self-injury, Paediatric, Emergency department, Young people

## Abstract

**Introduction:**

COVID-19 pandemic-related ‘lockdowns’ (i.e., measures to restrict movements) used to reduce the spread of the COVID-19 virus may have impacted how children and young people (CYP) utilised mental health services. The aim of this review was to synthesise research on COVID-19-related changes in mental health-related emergency department (ED) presentations by CYP in Australia, with reference to lockdown stringency.

**Methods and analysis:**

We searched five databases for observational studies published after 31/12/2019 examining ED mental health-related presentations by CYP (aged 0–24 years) before and after onset of COVID-19 lockdowns. Studies were stratified by location and the relative restriction stringency, obtained via the Oxford COVID-19 Government Response Tracker. Region level variation was described, with pooled effects calculated using random effects models.

**Results:**

Thirteen studies were included; five from New South Wales, four from Victoria (where lockdowns were most stringent), three from Queensland, and one including multiple Australian states. Outcome definitions and exposure periods were heterogeneous. Pooled estimates showed a moderate-to-large increase in monthly rates of eating disorder presentations, slight-to-moderate increases in overall mental health-related presentations and self-harm and suicide-related presentations, and inconclusive evidence for changes in substance-related and addictive disorder and behavioural/neurodevelopmental disorder-related presentations. Heterogeneity of pooled effects was extremely high (I^2^ = 84.8–99.5%), and the overall certainty of evidence was very low for all outcomes. Findings differed by jurisdiction, but there was insufficient evidence to identify an association between lockdown stringency and ED presentations.

**Conclusions:**

CYP ED presentations for eating disorders appear to have increased over the pandemic period, though certainty of evidence was very low. Findings for overall mental health-related presentations and other disorder groups were mixed, with presentations appearing to be lower in Queensland than other states. Future research should prioritise high quality interrupted-time-series analyses of linked and standardised administrative data to better examine the association between specific pandemic policy measures and CYP mental health-related ED utilisation, whilst controlling for potential confounders.

**Systematic review registration:**

This review has been registered with PROSPERO (CRD42022342496).

**Supplementary Information:**

The online version contains supplementary material available at https://doi.org/10.1186/s12873-026-01700-x.

## Introduction

The COVID-19 pandemic caused significant disruption to the lives of children and young people (CYP) [1–3]. Widespread school and university shutdowns and stay-at-home orders, commonly referred to as ‘lockdowns’, reshaped CYP’s social and educational environments and increased the digitisation of these interactions [4]. This may have been beneficial for some CYP [5] but for many others, social isolation, health anxiety, and socioeconomic pressures may have adversely impacted their mental health [6–9]. The restrictions may also have altered their access to – and use of – mental healthcare providers, potentially increasing reliance on emergency healthcare services [10].

There is evidence that COVID-19 public health policies were associated with changes in mental health-related emergency department (ED) presentations by CYP [11], including those aged 18–24 years [12] – with both increases and decreases reported [13–15]. Mental health-related ED presentations are not a direct measure of community level mental-health burden [16]. However, for the CYP who present, these ED presentations represent episodes of acute, often severe psychological distress [17] and may signal the onset of longer-term mental health difficulties. Whilst the most severe outcomes remain uncommon, ED mental health presentations are amongst the strongest predictors of later chronic mental illness and suicide death [18, 19]. These ED presentations also result in significant burden for CYP, their families, and healthcare systems [20]. Therefore, it is vital that any changes in these presentations during the COVID-19 pandemic period are identified and interrogated.

The COVID-19 pandemic provided an unprecedented opportunity to examine the unintended consequences of large-scale public health policy within a natural experiment framework, as governments worldwide implemented rapidly evolving restrictions with varying stringency and duration [21, 22]. However, within the rapidly evolving context of disease transmission and policy responses, disentangling the impacts of lockdown measures from those of the disease itself remains methodologically challenging [22]. In Australia, a state of emergency was declared in March 2020 and continued until October 2023, with most lockdown measures ceasing in late 2022 as vaccination targets were met [23, 24]. The stringent lockdown measures enacted by Australian federal and state governments resulted in some of the lowest COVID-19 mortality rates in the world [23]. However, the stringency and duration of lockdowns varied between states and territories in response to varying rates of infections and vaccine uptake [23, 24]. Australia’s two most populous cities, Sydney New South Wales (NSW) and Melbourne Victoria (VIC), experienced the most COVID-19 cases and, as a result, spent the longest time in lockdowns. In Melbourne, some of the longest and strictest lockdowns in the world were implemented over 2020 and 2021 [23]. Conversely, other geographically large states such as Queensland (QLD) and Western Australia (WA) opted to close their borders. These states had fewer COVID-19 cases and spent less time in lockdown [23, 24]. In combination, the relatively low burden of COVID-19 related illness and high stringency of policy responses in Australia provide an opportunity to examine the associations between lockdowns and mental health service utilisation, whilst potentially lowering the confounding effects of disease burden on population mental health and help-seeking behaviours [25]. Furthermore, the differing lockdown experiences across states allow for comparisons within a relatively homogenous social and health system context, sharing federal healthcare infrastructure and broadly similar demographic characteristics.

In anticipation of the potential secondary impacts of the pandemic and associated control measures, the Australian government introduced measures to mitigate economic and psychological stressors, including financial income support and job protection measures, an expansion of federally subsidised psychology treatment sessions, and targeted public awareness campaigns [23, 24]. These measures may have mitigated some mental health effects of lockdowns [26], particularly for adults [27]. Indirect support of this hypothesis was indicated by stable rates of suicide deaths in Australia during the pandemic [10], contrary to early predictions of an increase [28] and in spite of widely reported increases in psychological distress [10]. Economic support may have provided more direct relief to young people in the workforce or higher education. However, CYP living at home may have experienced indirect benefits through reduced household financial stress [29].

Several large studies have investigated the association between COVID-19 lockdowns and CYP mental health-related presentations to the ED. A cohort study of 23 hospital networks across 10 countries in the Eastern Mediterranean and European regions found that lockdown stringency was associated with increases in self-harm related ED presentations [30]. Several global systematic reviews of CYP mental health-related ED presentations reported that overall rates declined [11, 15, 31], whilst rates of self-harm and suicide-related presentations [11, 15] and eating disorders [31] increased. One of these reviews included a single Australian study [15], whilst the other two included five [11] and seven [31], respectively. For the latter two, an analysis of the moderating effect of geographic region for Australian studies was inconclusive for self-harm and suicide-related presentations [11], while there was moderate-to-strong evidence of an increase in ED presentations for eating disorders [31]. However, neither review attempted to systematically contextualise their findings using region-level factors which may have influenced increases in CYP mental health-related ED presentations, such as the stringency of lockdown policies or the level of economic support provided alongside these restrictions. Australia’s COVID-19 pandemic experience involved relatively high stringency of lockdown measures, low COVID-19 case numbers and deaths, and varied approaches in each state and territory. As such, examining the association between Australian lockdown stringency and mental health-related ED presentations by CYP may improve our understanding of the unintended consequences of restrictive pandemic policies and assist with planning for future pandemics.

The aims of our review are to [1] provide an updated synthesis of the evidence regarding the association between the COVID-19 pandemic and changes in mental health-related ED presentations by CYP in Australia; [2] compare changes in different states and territories according to the stringency of COVID-19 restrictions and levels of economic support; and [3] identify gaps in the literature and priority areas for future research.

## Methods and analysis

Our review conforms to the Preferred Reporting Items for Systematic Reviews and Meta-Analysis for Systematic Reviews (PRISMA) guidelines (Supplementary File A) [32] and the Meta Analysis of Observational Studies in Epidemiology (MOOSE) checklist (Supplementary File B) [33, 34]. The protocol has been registered with PROSPERO (CRD42022342496).

### Eligibility criteria

#### Participants

We included original research studies published in English that compared children and/or young people aged between 0 and 24 years who attended Australian EDs with primarily mental health-related presentations before and after the onset of the COVID-19 pandemic. Young people aged 18–24 years old were included as they are a high-risk population for ED attendance with mental health concerns [35], experience rapid changes in social roles and continued biological development, but are often excluded from research on adolescence [36]. We excluded studies relating to the COVID-19 pandemic where results from people aged < 25 years were unable to be independently extracted. Studies published prior to 31 December 2019 (when the Municipal Health Commission, China, identified the first cluster of pneumonia cases in Wuhan, later identified as COVID-19) [37] were excluded.

#### Exposure and outcome measures

Studies that investigated COVID-19 cases or government containment measures associated with the COVID-19 pandemic (e.g., school closures, stay-at-home orders) as a primary exposure were included. We also used data on lockdown severity sourced from the Oxford COVID-19 Government Response Tracker (OxCGRT), developed by researchers at the Blavatnik School of Government, University of Oxford [38]. The OxCGRT collected national and subnational data on government policy measures aimed at reducing the spread of COVID-19 across 180 countries. These data are coded into 23 indicators (e.g., vaccination policy, school closures, travel restrictions), that are scaled according to the extent of government action. Scores are aggregated into indices that represent different areas of policy (e.g., economic relief, containment, and closures) and range from 0 to 100, with higher scores indicating greater levels of government intervention. Our study utilised the stringency index (which describes the strictness of lockdown policies which restrict people’s behaviour), and the economic support index (which summarises measures such as debt relief and income support) [38]. We retrieved daily stringency and economic support index scores and used the city, state, or territory in which each study was conducted to determine the average daily scores for lockdown OxCGRT stringency and economic support during each study’s observation periods. No restrictions were placed on the length of the exposure or control periods of studies; however, OxCGRT coverage was limited to the period of March 2020 to March 2023 [38].

Our primary outcome measure was the monthly rate of overall mental health-related presentations to the ED (total mental health-related, as defined by study authors). In line with previous systematic reviews [17, 39], mental health-related presentations were defined as those with a primary diagnosis of one or more mental, neurodevelopmental, and/or behavioural disorders, and/or suicidal ideation, and/or intentional self-harm. Secondary outcomes included presentations associated with specific mental health-related diagnoses/presenting complaints, including:

- Self-harm and suicide-related presentations.
- Depressive/mood disorders.
- Anxiety disorders.
- Behavioural/neurodevelopmental disorders.
- Substance-related/addictive disorders.
- Eating disorders.
- Personality disorders.
- Schizophrenia spectrum and other psychotic disorders.

Presenting complaints and diagnostic categories examined in included studies were grouped within the above categories. As all studies were drawn from the same national health system, no restriction was placed on how included studies defined mental health-related presentations.

### Study design

This review considered observational studies including cohort, case-control, and cross-sectional studies, before-and-after time series, and interrupted time series studies. Qualitative studies, case reports, case studies, opinions, editorials, commentaries, letters, conference abstracts, and book chapters were excluded. Previous reviews, including systematic reviews, were excluded.

#### Information sources and search strategy

An initial limited search of PubMed and Google Scholar was undertaken to identify studies on the topic. The research team and a research librarian from the Royal Children’s Hospital, Melbourne developed a full search strategy for Medline using the text words contained in the titles and abstracts of relevant studies and the index terms used to describe the studies (e.g., Australia, COVID-19, mental health, emergency medical services, adolescent; see Supplementary File C for the full search strategy). The lead author adapted the search strategy with assistance from the librarian, including all identified keywords and index terms, for PsycINFO, PubMed, Embase, and CINAHL. The final search was conducted on 31 October 2024.

#### Study selection

Following the search, all identified studies were collated in Endnote X20 and uploaded into Covidence systematic review software [40], prior to duplicate removal. Both (1) title and abstract screening and (2) detailed full text screening of selected studies against the inclusion criteria were conducted in Covidence by two independent reviewers. Agreement was moderate in both title and abstract (κ = 0.78, 91% agreement) and full text (κ = 0.68, 84% agreement) screening [41]. Disagreements between reviewers were resolved through discussion or with assistance from an additional reviewer.

#### Data extraction

Data extraction from the studies was conducted in Covidence by a single author, using a pre-specified extraction form developed by the study team (Supplementary File D). The following characteristics were extracted from eligible studies: author(s), year of publication, study location(s), year(s) and months of study, study design, sample selection techniques (e.g., whole target population vs. selected samples such as children, adolescents, or those with specific mental disorders), demographic characteristics, data source(s) (e.g., hospital administrative patient records, administrative database), cohort size, and comparison group(s). OxCGRT data related to index scores on restriction stringency and economic support were then extracted for the exposure period of each study. All extracted information was double-checked for accuracy, prior to analysis.

#### Certainty of evidence and risk of bias assessment

Certainty of evidence was assessed for each pooled outcome across all five Grading of Recommendations Assessment, Development and Evaluation (GRADE) framework domains (risk of bias, inconsistency, indirectness, imprecision, publication bias) [42] using GRADEpro [43], and results were summarised in a GRADE evidence profile table (Supplementary File E). Assessment was conducted by one reviewer rather than two independent reviewers, assumed risk was not calculated due to the rate-based analytical approach used in this review, and the assessment was not pre-specified in the registered protocol. Certainty ratings should therefore be interpreted with these limitations in mind.

To assess risk of bias of included studies, we utilised the Methodological Standard for Epidemiological Research (MASTER) scale [44]. The MASTER scale assesses the risk of bias in studies by the presence of 36 items/methodological safeguards grouped into six potential bias domains (selection, information, design-related, analytic, confounding, and external validity) [44, 45]. A total score is derived from the number of safeguards that are present and applicable to the design of each included study. A relative risk of bias score was generated by taking the highest scoring study included in our review and calculating the relative proportion of the number of safeguards met by other studies [46, 47]. For example, if the highest scoring study had 20 bias safeguards present, it would receive a relative risk of bias score of 100 and a study with 16 bias safeguards would receive a relative risk of bias score of 80. The relative risk of bias score means that bias safeguards that are inapplicable to all studies in a review (for example, those related to randomised control trials for the present review) do not unfairly penalise studies. Two assessors evaluated all studies, with moderate agreement (κ = 0.78, 86% agreement) across bias domains [41]. Discrepancies were resolved through discussion.

### Data synthesis

Included studies were summarised in a tabular format. To address heterogeneity of denominators used in adjusted and unadjusted effect estimates reported within studies, the monthly rate of ED presentations in the pre-COVID-19 control group and COVID-19 exposure group were extracted for each included study to calculate monthly presentation rate ratios. If not directly reported, monthly rates were calculated by dividing the total control and exposure period presentation counts by the number of months in each period. Where insufficient data were available to calculate cases in the pre-COVID-19 and COVID-19 groups, a maximum of two requests for additional data were sent to study authors. If no response was received after two months, it was assumed that no data would be forthcoming. If a study had multiple time-points in either the pre-COVID-19 (e.g., data reported separately for 2018 and 2019) or COVID-19 (e.g., first lockdowns, lockdown eased period, or second lockdown) periods, these were combined. If combination was not possible, e.g., there was insufficient information to extrapolate total presentations from reported rates and total time-period in each discrete COVID-19 time-period, only the single longest of reported pre-COVID-19 and/or COVID-19 control/exposure windows were included. The overall monthly rate ratio (RR) and 95% confidence interval (CI) of mental health-related ED presentations before and after the onset of the COVID-19 pandemic were generated for the primary outcome (overall mental health-related presentations) and five secondary outcomes (self-harm and suicide-related, anxiety disorder, behavioural/ neurodevelopmental disorder, substance-related and addictive disorder, and eating disorder presentations), and presented graphically (see Fig. 2). Regional patterns in ED utilisation were described based on these calculated monthly rate ratios rather than study-reported effect estimates, to ensure comparability across studies using heterogeneous analytical approaches. Effect estimates calculated for each individual study are described as either (a) increases (95% CI entirely above 1.00), (b) decreases (95% CI entirely below 1.00), or (c) no change (95% CI crosses 1.00).

Where studies had crossover in recruitment across geographic location, inclusion criteria, and study period, they were deemed likely to have recruited the same or overlapping patients. In each case where this occurred, studies were compared in terms of sample size, exposure period, and geographic scope, and the smaller or less representative study was excluded from pooled analysis. A detailed description of the decision making for study removal and retention can be found in Supplementary Table S3. After removal of studies with overlapping patient populations, calculated monthly presentation rate ratios were pooled using a random-effects meta-analysis in Stata 18 [48]. A random-effects model was used to account for expected heterogeneity across studies, given anticipated differences in disease transmission, lockdown stringency, health system capacity, and population characteristics between Australian states and territories [49]. Pooled effects were generated separately for:

1. Overall mental health-related presentations.
2. Self-harm and suicide-related presentations.
3. Behavioural/neurodevelopmental disorder presentations.
4. Eating disorder presentations.
5. Substance-related/addictive disorder presentations.

Effect sizes of pooled rate ratio confidence limit increases and decreases (95% CI < > 1.00) were interpreted within predefined thresholds developed by Madigan et al. [11], corresponding to clinically significant changes in patient visit numbers:

- Decreases
  - large (< 0.50)
  - moderate (0.50 to < 0.70)
  - small (0.70 to < 0.90)
  - slight (0.90 to < 1.00)

- Increases
  - slight (1.00 to < 1.11)
  - small (1.11 to < 1.43)
  - moderate (1.43 to < 2.00)
  - large (≥ 2.00)

- Inconclusive
  - Unable to determine as CI crosses unity (both above and below 1.00)

Publication bias was assessed using funnel plots and corresponding Egger’s tests [50] and heterogeneity was assessed using *I*^*2*^ statistics. As there was an insufficient number of included studies to perform a meta-regression using region-level indicators (COVID-19 pandemic-related government restrictions and economic support), we presented data graphically alongside summary statistics on region-level stringency measures and ED presentations.

## Results

**Fig. 1:**
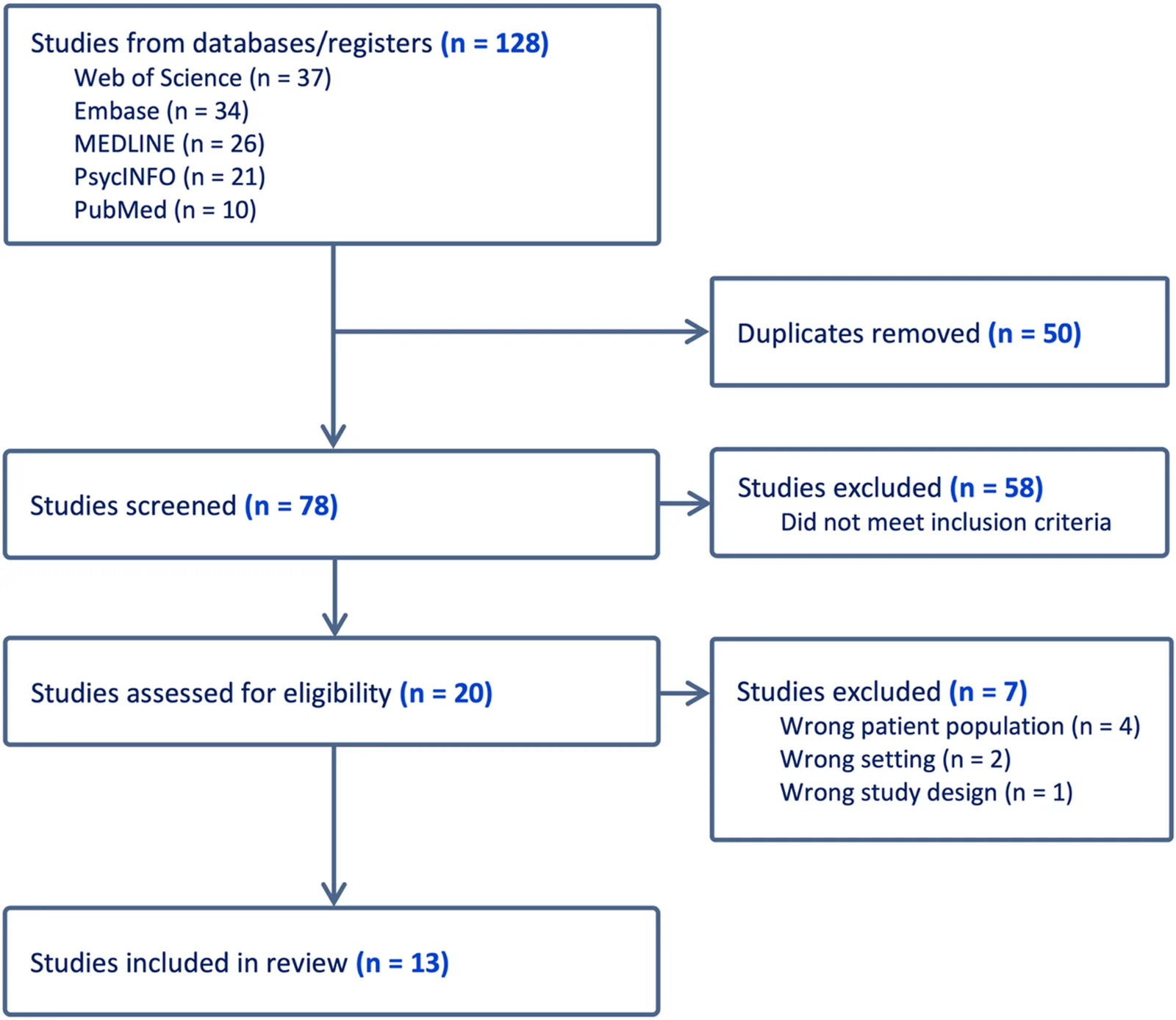
PRISMA flow diagram of the systematic search and screening process of potentially eligible studies

Seventy-eight non-duplicate records were identified within our search, with 20 full texts reviewed for eligibility. After all exclusions, 13 studies were included in the review (Fig. 1) [12, 35, 51–61]. The seven studies excluded after full text review are described in Supplementary Table S1.

### Study characteristics

Of the 13 included studies, five were from the state of New South Wales (NSW) [35, 53, 55, 58, 59], four from Victoria (VIC) [12, 51, 52, 54], three from Queensland (QLD) [56, 60, 61], and one included data from the capital cities of five states (NSW, VIC, QLD, Western Australia [WA], and South Australia [SA]) [57] (Table 1). Weighted against the highest score of 23 (64% of total possible safeguards), the quality and relative risk of bias scores of included studies ranged from 65 to 100, with a median of 91 (IQR [83,96]), indicating generally high quality for retrospective cohort designs. Five studies received the median score, whilst four scored higher (indicating lower risk of bias) and four scored lower (Supplementary Figure S1; Supplementary Table S2). Those scoring below the median value had lower scores for equal retention [51, 52, 59], equal ascertainment [52, 61], equal implementation [61], equal prognosis [51, 52], sufficient analysis [61], and temporal precedence [51, 52, 59]. Eight studies included analysis of overall mental health-related presentations [12, 51, 52, 54–57, 61], with six disaggregating this into specific diagnostic categories (including self-harm and suicide-related presentations) [12, 51, 54, 56, 57, 61]. Five studies looked exclusively at self-harm and suicide-related presentations [35, 53, 58–60]. Nine studies examined COVID-19-related presentations in 2020 [12, 51, 52, 54, 56, 58–61], whilst the remaining four included data from 2020 to 2021 [35, 53, 55, 57]. Whilst diagnostic inclusion criteria were broadly similar, the grouping of specific diagnoses within outcome categories varied between studies – for example, some studies grouped depression and anxiety separately, whilst others grouped them together or used broader categories such as mood disorders, and definitions of self-harm and suicide-related presentations varied in their inclusion of suicidal ideation, self-harm, and suicide attempts (Supplementary File F).

Across all 13 studies, a combined sample of 309,114 mental health-related ED presentations was identified. For one study [59], sample size could not be determined, and authors did not respond to data requests within the specified timeframe. Review of studies with potential overlapping study populations (Supplementary Table S3) resulted in the removal of four studies for overall mental health-related presentations [51, 52, 57, 61]; five for self-harm, suicide attempt and suicidal ideation [51, 53, 57–59]; two for neurodevelopmental and behavioural disorders [51, 57], two for eating disorders [51, 57]; one for substance-related and addictive disorders [57]; and two for anxiety disorders [51, 57]. Following all removals, the revised total sample for meta-analysis was 233,914 unique ED visits, across seven studies.

**Fig. 2:**
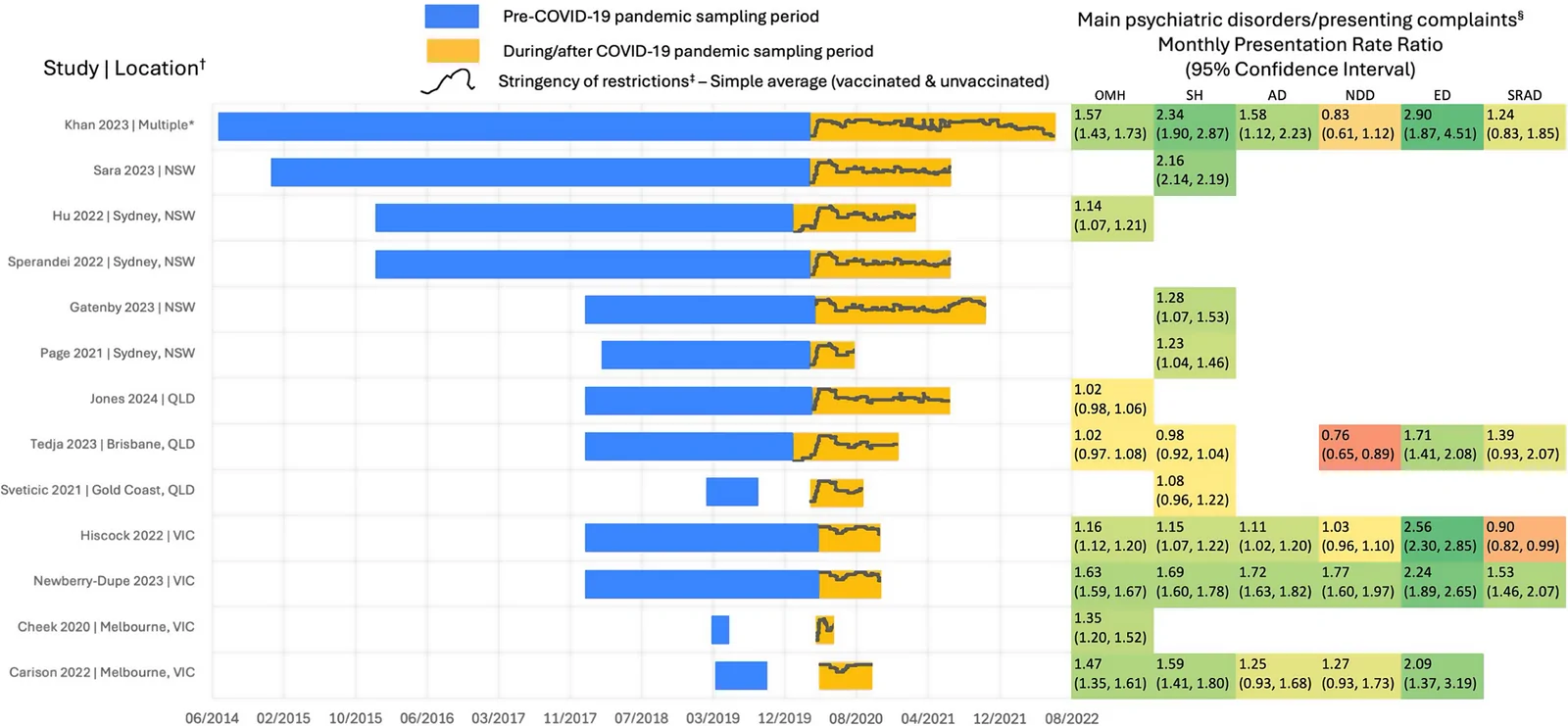
Control and exposure sampling windows, COVID-19 restriction stringency, and relative changes in monthly rates of mental health-related ED presentations. Green shading of risk ratios indicates higher presentation rates in the COVID-19 period, yellow means no difference, and red means lower rates during the COVID-19 period. ^†^Study names are shortened: ‘et al.’ removed to preserve width (see Table 1); locations are abbreviated: NSW = New South Wales; QLD = Queensland; VIC = Victoria. ^‡^As measured by the Oxford Government Response Tracker Stringency Index, a composite score from 0 to 100 rating the stringency of government policies to prevent community transmission of COVID-19. Scores represent the simple daily average of rules which applied to vaccinated and non-vaccinated people [38]. ^§^Included psychiatric disorders/presenting complaints: OMH = overall mental health-related presentations, SH = self-harm, suicide attempt and suicidal ideation, AN = anxiety disorders, NDD = neurodevelopmental and behavioural disorders, ED = eating disorders, SRAD = substance-related and addictive disorders, *Multiple sites = Sydney, NSW; Perth, WA; Melbourne, VIC; Brisbane, QLD; Adelaide, SA

Figure 2 summarises the control and exposure periods for each study, the stringency of COVID-19 restrictions (as measured by the OxCGRT stringency index), and the ratio of monthly visit rates before/during the COVID-19 pandemic period. Rate ratios are shown for overall mental health-related presentations and the five most frequently disaggregated presenting diagnoses/concerns across studies.

The stringency of COVID-19 restrictions varied between locations and across the varying lengths of exposure periods of included studies. Conversely, economic support (as measured by the OxCGRT economic support index, which primarily captured federal economic support policies) was largely consistent across locations (Table 1). Stringency was notably elevated for the sampling windows of VIC studies compared to other locations (Table 1), although these windows were all within the first 12 months of the pandemic (Fig. 2). The pattern of stringency was broadly similar in QLD and NSW studies (Table 1; Fig. 2).

### Descriptive summary of regional differences

Of the eight studies reporting overall mental health-related presentations, an increase in the calculated monthly rate ratios was identified for six (75%) [12, 51, 52, 54, 55, 57], whilst no change was found in two (25%) [56, 61]. Studies where an increase was found were located in VIC [12, 51, 52, 54], NSW [55], and multistate [57], whilst those with no change were in QLD [56, 61]. Similarly, of the nine studies which reported self-harm and suicide-related presentations, an increase was found for seven (78%) [12, 35, 51, 53, 54, 57, 58], with no change found for two (22%) [60, 61]. Those with increases were in VIC [12, 51, 54], NSW [35, 53, 58], and multistate [57], whilst those with no change were in QLD [60, 61]. Increases in anxiety-related presentations were found for two of three studies in VIC [12, 54] and one multistate study [57], with no change for one VIC study [51]. Increases in eating disorder presentations were found in VIC [12, 51, 54], QLD [61], and multistate [57] studies. Changes in neurodevelopmental and behavioural disorder presentations were mixed, with a decrease in QLD [61], no change in the multistate study [57], no change in two VIC studies [51, 54], and an increase in young people aged 18–24 years in one VIC study [12]. Findings for substance-related and addictive disorders were similarly mixed, with a decrease in those aged < 18 years [54] and an increase for those aged 18–24 years [12] in VIC studies, and no change in QLD [61] and multistate [57] studies.

**Table 1:** Characteristics of included studies

	Location^†^	Age of participants^‡^ (years)	Visit types included^¶^	Sample size	Data collection windows		OxCGRT Stringency Index (mean)	OxCGRT Economic Support Index (mean)
					Before COVID-19	COVID-19		
Carison et al. (2022) [51]	VIC	7–18	Overall mental health-related presentations Anxiety disorders; self-harm; suicide attempt; suicidal ideation; disruptive, impulse-control, and conduct disorders; eating disorders; other	1,999	1 Apr – 30 Sep 2019	1 Apr – 30 Sep 2020	84.7	62.1
				-				
Cheek et al. (2020) [52]	Melbourne, VIC	0–17	Overall mental health-related presentations	1,141	20 Mar – 20 May 2019	20 Mar – 20 May 2020	84.1	54.4
Gatenby et al. (2023) [53]	NSW	0–16	Self-harm/Suicide attempt (poisoning)	501	1 Jan 2018–22 Mar 2020	23 Mar 2020–31 Oct 2021	63.1	63.4
Hiscock et al. (2022) [54]	VIC	0–17	Overall mental health-related presentations Depressive/Mood disorders; anxiety disorders; self-harm; substance-related and addictive disorders; development/behavioural disorders; eating disorders	20,645	1 Jan 2018–30 Mar 2020	31 Mar 2020–28 Oct 2020	83.0	58.5
				-				
Hu et al. (2022) [55]	NSW	0–17	Overall mental health-related presentations	5,437	1 Jan 2016–31 Dec 2019	1 Jan 2020–28 Feb 2021	50.9	53.6
Jones et al. (2024) [56]	QLD	5–17	Overall mental health-related presentations Anxiety disorder; Psychotic disorder; Mood disorder; Intentional self-harm; Eating disorder; Other	19,291	1 Jan 2018–8 Mar 2020	9 Mar 2020–28 Jun 2020	60.4	63.4
				-				
Khan et al. (2023) [57]	Multiple: Sydney, NSW Perth, WA Melbourne, VIC Adelaide, SA Brisbane, QLD	0–18	Overall mental health-related presentations Depressive/Mood disorders; Anxiety disorders; Self-harm; Disruptive, impulse-control, and conduct disorders; Substance-related and addictive disorders; Trauma- and stressor-related disorders; Eating disorders; Other (Mental health symptoms)	70,260^§^	14 Jul 2014–28 Feb 2020	01 Mar 2020–31 Dec 2021	55.1	55.6
				-				
Newberry-Dupe et al. (2023) [12]	VIC	18–24	Overall mental health-related presentations Depressive/Mood disorders; Anxiety disorders; Self-harm; Personality disorders; Schizophrenia spectrum and other psychotic disorders; Substance-related and addictive disorders; Neurodevelopmental disorders; Eating disorders	33,851	1 Jan 2018–31 Mar 2020	1 Apr – 31 Oct 2020	82.7	58.3
				-				
Page et al. (2021) [58]	NSW	15–24	Self-harm	535	1 Jan – 31 Jul 2019	1 Jan – 31 Jul 2020	61.9	58.7
Sara et al. (2023) [35]	NSW	10–24	Combined suicidal ideation, self-harm & suicide attempt Self-harm (including suicide attempt); Suicidal ideation	148,008	1 Jan 2015–28 Feb 2020	1 Mar 2020–30 Jun 2021	57.0	64.5
Sperandei et al. (2022) [59]	NSW	0–24	Self-harm, suicide attempt, and/or ideation Self-harm; Suicide attempt; Suicidal / self-harm ideation	-	1 Jan 2016–28 Feb 2020	1 Mar – 30 Jun 2021	63.0	57.6
Sveticic et al. (2021) [60]	Gold Coast, QLD	0–17	Combined suicidal ideation, self-harm & suicide attempt	1,034	1 Mar – 31 Aug 2019	1 Mar – 31 Aug 2020	64.7	77.0
Tedja et al. (2023) [61]	Brisbane, QLD	5–18	Overall mental health-related presentations Anxiety & Depression; Self-harm; Suicidal ideation/attempt; Suicide threats; Disruptive, impulse-control, and conduct disorders; Schizophrenia spectrum and other psychotic disorders; Substance-related and addictive disorders; Eating disorders; Other	6,412	1 Jan 2018–31 Dec 2019	1 Jan – 31 Dec 2020	52.0	56.2
				-				

† Locations abbreviated: NSW = New South Wales; VIC = Victoria; QLD = Queensland; WA = Western Australia; SA = South Australia

‡ Age of study participants < 25 years. Excludes study participants aged > 24 years

¶ Visit types were aggregated into eight pre-defined categories according to best fit. Actual visit type category names varied more substantially between studies, see Supplementary File F

§ Estimated sample size. Total sample across pre-COVID-19, COVID-19, and COVID-19 restriction-eased (1 Jan– 30 Jun 2022) periods was 75,894. Average monthly presentations during the COVID-19 restriction-eased period 939.0. Sample size for pre-COVID-19 and COVID-19 periods was estimated using the formula: total sample – (COVID-19 restriction-eased period average monthly presentations x 6 months)

### Changes in ED utilisation for psychiatric disorders/presenting complaints

**Fig. 3:**
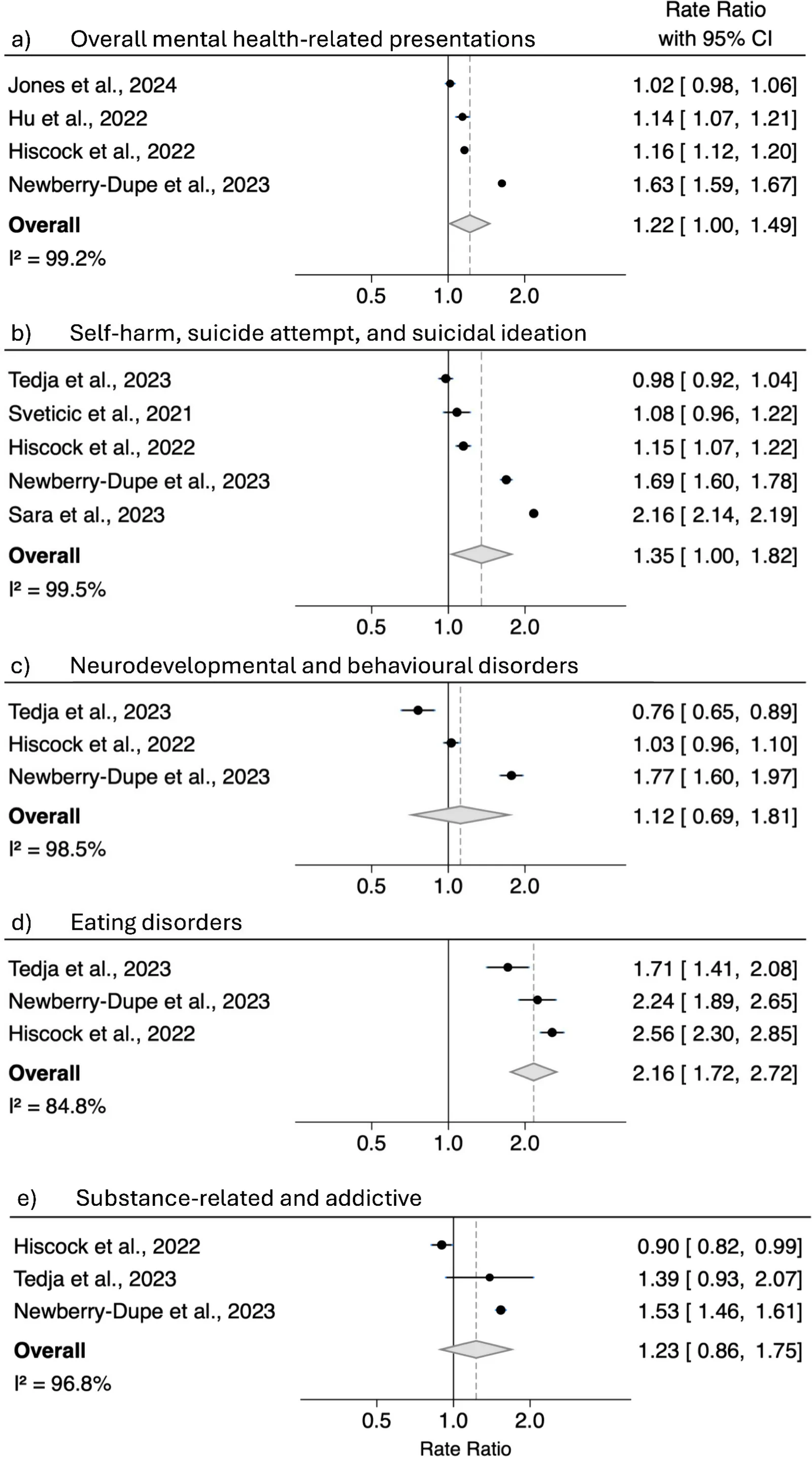
Changes in monthly rate of child and young person visits to ED for (**a**) overall mental health-related concerns, (**b**) self-harm, suicide attempt, and suicidal ideation, (**c**) neurodevelopmental and behavioural disorders, (**d**) eating disorders, and (**e**) substance-related and addictive disorders during the COVID-19 pandemic period compared to before the pandemic

Figure 3 presents the relative risk of children and young people presenting to the ED before and during the COVID-19 pandemic period for (a) overall mental health-related presentations, including self-harm; (b) self-harm, suicide attempts, and suicidal ideation; (c) neurodevelopmental and behavioural disorders; (d) eating disorders; and (e) substance-related and addictive disorders. For each disorder/presenting complaint group, a random effects meta-analysis was conducted and overall effect estimates derived.

Meta-analyses identified a slight to small increase in ED presentations for overall mental health-related presentations (RR 1.22, 95% CI [1.00, 1.49], I^2^ = 99.2%) and a slight to moderate increase for self-harm, suicide attempts, and suicidal ideation (RR 1.35, 95% CI [1.00, 1.82], I^2^ = 99.5%). There was a moderate to large increase for eating disorder presentations (RR 2.16, 95% CI [1.72, 2.72], I^2^ = 84.8%) with concordant direction and magnitude across contributing studies (Fig. 3), and inconclusive evidence for changes in visits for neurodevelopmental and behavioural disorders (RR 1.12, 95% CI [0.69, 1.81], I^2^ = 98.5%) and substance-related and addictive disorders (RR 1.23, 95% CI [0.86, 1.75], I^2^ = 96.8%). In all meta-analyses, between-study heterogeneity was extremely high (I^2^ range = 84.8–99.5%) and the GRADE certainty of evidence was very low (Supplementary File E), indicating that pooled estimates should be interpreted with caution.

### Moderating factors of changes in ED utilisation for mental health concerns

Only five (38%) of all included studies reported differences in mental health-related presentations by gender in participants aged under 25 years. Too few studies were included to conduct subgroup analyses – however, an exploratory narrative synthesis of the five studies indicated that women and girls were reported to have presented at disproportionately increased rates for eating disorders [12] and self-harm and suicide [35, 58] during the COVID-19 pandemic exposure period compared to pre-COVID-19 control periods, compared to men and boys. Conversely, reported changes for overall mental health-related presentations by women and girls were mixed, with two studies reporting increases [51, 57] and one reporting a decrease [61].

## Discussion

In this review, we measured the association between changes in mental health-related ED presentations by CYP aged 0–24 years, the stringency of lockdowns, and the level of economic support enacted by Australian national and state governments over the COVID-19 pandemic period. In line with previous research [31], our meta-analysis identified a two-fold increase in eating disorder presentations to Australian EDs, most notably in Victoria (VIC) in 2020. Broadly, identified changes in overall mental health-related presentations and self-harm and suicide-related presentations were mixed, and there was inconclusive evidence regarding changes in presentations by CYP with concerns relating to neurodevelopmental and behavioural disorders or substance-related and addictive disorders. Heterogeneity was extremely high for all outcomes (I² range = 84.8–99.5%). Given the observational and retrospective design of included studies, high between-study heterogeneity, and limited number of studies per outcome, the overall GRADE certainty of evidence was very low [42, 62]. Eating disorder presentations represented the most consistent finding, with a moderate-to-large pooled effect and concordant direction and magnitude of changes across contributing studies. With regards to overall mental health-related presentations and self-harm and suicide-related presentations, increases were reported in VIC, NSW and multistate studies, but no change was reported in studies from QLD alone. Whilst the stringency of restrictions was notably higher in VIC, there was little difference in restriction stringency in QLD and NSW during the exposure periods of included studies, despite differences in reported increases in ED mental health-related presentations between studies conducted in these two states. This could indicate that factors beyond lockdown stringency contributed to interstate differences, including variability in diagnostic coding practices, differences in mental health service structure and accessibility [63], or differences in COVID-19 transmission burden between states [64]. Therefore, whilst it remains possible that the high stringency in VIC may have been associated with elevated presentations for mental health-related concerns, further research is required to determine whether other factors were associated with reported increases, or whether the stringency of specific policies and restrictions were associated with increasing ED mental health-related presentations by CYP. Future studies which conduct interrupted time-series analyses using linked administrative service utilisation data from Australian state, territory, and federal health services would have greater capacity to examine associations between policy responses and changes in ED utilisation whilst controlling for potential confounders [65]. Future systematic reviews could improve comparability by restricting inclusion to studies using consistent diagnostic classification systems and comparable exposure period definitions, reducing between-study heterogeneity. With a sufficient body of high-quality interrupted time-series studies, future reviews could provide greater certainty regarding state-level policy differences and other potential contributors to changes in ED utilisation, including service capacity differences and local COVID-19 case burden.

The next global pandemic is considered an inevitability [66, 67], and the World Health Organization and other international health bodies have begun identifying plans to mitigate its effects [67]. Given their effectiveness at controlling disease transmission [23], it is likely that tools such as lockdowns will remain a central element of future pandemic response strategies. Both the COVID-19 pandemic itself and associated preventative measures have been associated with changes in the mental health and mental health service utilisation patterns of some CYP [64, 68]. From the early stages of the pandemic, consistent concerns were raised about the potential negative impacts on CYP mental health [28]. Universal increases in the prevalence of mental disorders did not appear to occur to the extent predicted [16]; however, our findings add to international evidence of an increase in the prevalence or severity of eating disorder-related presentations to ED by CYP after the onset of the pandemic [31, 68]. Our findings are indicative of eating disorder-related service utilisation rather than community-level prevalence. However, studies of community samples appear to support a concurrent rise in reported eating disorder symptoms among CYP in Australia [69] and internationally [70]. Eating disorders, particularly cases of Anorexia Nervosa requiring hospitalisation, can have lifelong consequences on health and wellbeing [71], and are associated with an elevated risk of early mortality [71, 72]. Therefore, policymakers preparing for a future pandemic must ensure their planning is informed by high quality evidence which accounts for the potential risks to CYP mental health and wellbeing. It remains unclear to what extent changes in CYP mental wellbeing and mental health service utilisation during the COVID-19 pandemic period were temporary, were reflective of changes in mental health symptoms or shifts in public messaging and broader service usage patterns, and whether there may be longer-term changes in the post-pandemic period. To ensure that these changes can be accurately understood, ongoing monitoring of the long-term trends in mental health symptoms and service utilisation patterns of CYP is required. Future research should compare and incorporate trends during the COVID-19 pandemic period with broader trends over time, using linked service utilisation data to assess long-term implications of pandemic-related changes for health systems as a whole, and identify whether trends have returned to a pre-pandemic trajectory [65].

### Strengths and limitations

To our knowledge, this is the first systematic review to examine the association between mental health-related ED presentations by CYP and lockdown stringency at the subnational level, and the first to examine this relationship in Australia. The review adhered to the PRISMA 2020 guideline [32] and the MOOSE checklist [33, 34] to improve transparency and accuracy. All screening was conducted by two authors, with discrepancies resolved through discussion. Furthermore, smaller/lower quality studies which were deemed to have any cross-over in recruitment location and timeframe with larger/more robust studies were excluded from the meta-analysis, to reduce the risk of multicollinearity on risk ratio coefficients.

Our review contained four main limitations. First, there was significant heterogeneity of results between included studies across all meta-analyses conducted (I^2^ >75), limiting the clinical applicability and generalisability of overall effect sizes. With insufficient study numbers to conduct the planned sub-group analyses, formal investigation of between-group differences was likely to be underpowered and potentially unreliable, further limiting interpretation of pooled estimates. The clinical significance of statistically significant pooled effects is therefore difficult to interpret. Second, comparability between included studies was limited by heterogeneous outcome definitions and substantial variability of exposure periods [73]. Diagnostic groupings varied – including differential definitions and inclusion of suicidal ideation, self-harm, and suicide attempt-related presentations between studies. Diagnostic practices may also have varied between hospitals included in studies. These factors likely contributed to between-study heterogeneity and limit the comparability of findings across outcomes and regions. Third, since a limited number of eligible studies were identified, we were unable to perform a planned meta-regression to assess the association of the stringency of restrictions, level of economic support, and rates of COVID-19 cases and deaths with changes in mental health-related ED presentations by CYP in Australia. As fewer than 10 studies were included in the meta-analysis, the reliability of publication bias assessments was limited [74], making it difficult to infer the presence or absence of publication bias in the included study pool and precluding reporting of Egger’s test outcomes. Future research could apply this research question to international studies to obtain sufficient study numbers and inter-region heterogeneity of restrictions and financial support to perform a meta-regression of the association between COVID-19 related policies and CYP mental health-related presentations and to enable reliable assessment of publication bias. Fourth, averaging over long exposure periods may have diluted the effect size of short, intense “snap” lockdowns which were interspersed with periods of relatively low stringency of restrictions [23, 24]. Where data are available, future studies should compare periods of high restrictions with periods where restrictions were eased *during* the COVID-19 pandemic period. All included studies utilised observational retrospective designs and administrative data, precluding causal inference. As a result of these and the methodological limitations described above, the overall GRADE certainty of evidence was very low [42], limiting the strength of conclusions that can be drawn. Implications of this study should be considered exploratory and hypothesis generating rather than definitive.

## Conclusions

In Australia, observational evidence for changes in mental health-related ED presentations by CYP during the COVID-19 pandemic period is mixed and highly heterogeneous. However, the most consistent finding provided evidence for increases in presentations by CYP with eating disorders. Given the medical complications associated with eating disorder presentations to hospitals, this potential increase could indicate a significant escalation of medical instability in this cohort, with potential long-term consequences on health and wellbeing. Continued monitoring of the CYP who experienced declines in mental health and wellbeing over the COVID-19 pandemic period is recommended as they transition to adulthood, to assess the long-term implications of reported increases in acute distress and ED utilisation on future health service utilisation and mental health outcomes.

## Supplementary Information

Below is the link to the electronic supplementary material.

Supplementary Material 1

Supplementary Material 2

## Data Availability

Researchers wishing to undertake additional analysis of the data extracted for this review are invited to contact the corresponding author. All data and STATA code will be made available upon reasonable request.

## Declarations

### Deviations from protocol

The scope of the original systematic review was narrowed from an international focus to an Australian focus, to generate targeted evidence for the Australian context. Within this limited scope, a full meta-regression was not feasible. As such, findings regarding the stringency of lockdowns are summarised in narrative format. Meta-analysis of a limited subset of included studies was conducted, due to the potential cross-over of patients included in studies conducted in the same locality.

## Abbreviations

CI: Confidence interval
COVID-19: Coronavirus disease 2019
CYP: Children and young people
ED: Emergency department
GRADE: Grading of Recommendations Assessment, Development, and Evaluation
MASTER: Methodological Standards for Epidemiological Research
MOOSE: Meta-analyses Of Observational Studies in Epidemiology
NSW: New South Wales
OxCGRT: Oxford COVID-19 Government Response Tracker
PRISMA: Preferred Reporting Items for Systematic reviews and Meta-Analyses
PROSPERO: International Prospective Register of Systematic Reviews
QLD: Queensland
RR: Rate ratio
VIC: Victoria
WA: Western Australia
