# Supplementary material for: Mental health-related presentations to Australian hospital emergency departments by children and young people, before and after the onset of the COVID-19 pandemic: a systematic review and meta-analysis
Source: BMC Emerg Med. 2026 Jul 22;26:277. doi: 10.1186/s12873-026-01700-x (PMC13685287; doi:10.1186/s12873-026-01700-x)
Supplement: Supplementary file 1 [file 12873_2026_1700_MOESM2_ESM.docx]

Appendices

**Supplementary File A**: PRISMA Checklist

| **Section and Topic** | **Item #** | **Checklist item** | **Location where item is reported** |
| --- | --- | --- | --- |
| **TITLE** | | |  |
| Title | 1 | Identify the report as a systematic review. | Page 1 |
| **ABSTRACT** | | |  |
| Abstract | 2 | See the PRISMA 2020 for Abstracts checklist. | Page 2 |
| **INTRODUCTION** | | |  |
| Rationale | 3 | Describe the rationale for the review in the context of existing knowledge. | Page 4-6 |
| Objectives | 4 | Provide an explicit statement of the objective(s) or question(s) the review addresses. | Page 6 |
| **METHODS** | | |  |
| Eligibility criteria | 5 | Specify the inclusion and exclusion criteria for the review and how studies were grouped for the syntheses. | Page 6 |
| Information sources | 6 | Specify all databases, registers, websites, organisations, reference lists and other sources searched or consulted to identify studies. Specify the date when each source was last searched or consulted. | Page 8 |
| Search strategy | 7 | Present the full search strategies for all databases, registers and websites, including any filters and limits used. | Supplementary File C |
| Selection process | 8 | Specify the methods used to decide whether a study met the inclusion criteria of the review, including how many reviewers screened each record and each report retrieved, whether they worked independently, and if applicable, details of automation tools used in the process. | Page 6-8 |
| Data collection process | 9 | Specify the methods used to collect data from reports, including how many reviewers collected data from each report, whether they worked independently, any processes for obtaining or confirming data from study investigators, and if applicable, details of automation tools used in the process. | Page 8-9 |
| Data items | 10a | List and define all outcomes for which data were sought. Specify whether all results that were compatible with each outcome domain in each study were sought (e.g. for all measures, time points, analyses), and if not, the methods used to decide which results to collect. | Page 6-8 |
|  | 10b | List and define all other variables for which data were sought (e.g. participant and intervention characteristics, funding sources). Describe any assumptions made about any missing or unclear information. | Page 6-8, Supplementary File D |
| Study risk of bias assessment | 11 | Specify the methods used to assess risk of bias in the included studies, including details of the tool(s) used, how many reviewers assessed each study and whether they worked independently, and if applicable, details of automation tools used in the process. | Page 9 |
| Effect measures | 12 | Specify for each outcome the effect measure(s) (e.g. risk ratio, mean difference) used in the synthesis or presentation of results. | Page 9-11 |
| Synthesis methods | 13a | Describe the processes used to decide which studies were eligible for each synthesis (e.g. tabulating the study intervention characteristics and comparing against the planned groups for each synthesis (item #5)). | Page 9-10 |
|  | 13b | Describe any methods required to prepare the data for presentation or synthesis, such as handling of missing summary statistics, or data conversions. | Page 9-10 |
|  | 13c | Describe any methods used to tabulate or visually display results of individual studies and syntheses. | Page 9, 11 |
|  | 13d | Describe any methods used to synthesize results and provide a rationale for the choice(s). If meta-analysis was performed, describe the model(s), method(s) to identify the presence and extent of statistical heterogeneity, and software package(s) used. | Page 10-11 |
|  | 13e | Describe any methods used to explore possible causes of heterogeneity among study results (e.g. subgroup analysis, meta-regression). | Page 6-7, 11 |
|  | 13f | Describe any sensitivity analyses conducted to assess robustness of the synthesized results. | NA |
| Reporting bias assessment | 14 | Describe any methods used to assess risk of bias due to missing results in a synthesis (arising from reporting biases). | Page 11 |
| Certainty assessment | 15 | Describe any methods used to assess certainty (or confidence) in the body of evidence for an outcome. | Page 9, Supplementary File E |
| **RESULTS** | | |  |
| Study selection | 16a | Describe the results of the search and selection process, from the number of records identified in the search to the number of studies included in the review, ideally using a flow diagram. | Page 11-12 |
|  | 16b | Cite studies that might appear to meet the inclusion criteria, but which were excluded, and explain why they were excluded. | Supplementary Table S1 |
| Study characteristics | 17 | Cite each included study and present its characteristics. | Page 12-16 |
| Risk of bias in studies | 18 | Present assessments of risk of bias for each included study. | Page 12, Supplementary Figure S1, Supplementary Table S2 |
| Results of individual studies | 19 | For all outcomes, present, for each study: (a) summary statistics for each group (where appropriate) and (b) an effect estimate and its precision (e.g. confidence/credible interval), ideally using structured tables or plots. | Page 15-18 |
| Results of syntheses | 20a | For each synthesis, briefly summarise the characteristics and risk of bias among contributing studies. | Page 19 |
|  | 20b | Present results of all statistical syntheses conducted. If meta-analysis was done, present for each the summary estimate and its precision (e.g. confidence/credible interval) and measures of statistical heterogeneity. If comparing groups, describe the direction of the effect. | Page 19-21 |
|  | 20c | Present results of all investigations of possible causes of heterogeneity among study results. | Page 12-14, 19 |
|  | 20d | Present results of all sensitivity analyses conducted to assess the robustness of the synthesized results. | NA |
| Reporting biases | 21 | Present assessments of risk of bias due to missing results (arising from reporting biases) for each synthesis assessed. | NA |
| Certainty of evidence | 22 | Present assessments of certainty (or confidence) in the body of evidence for each outcome assessed. | Page 19, Supplementary File E |
| **DISCUSSION** | | |  |
| Discussion | 23a | Provide a general interpretation of the results in the context of other evidence. | Page 21-23 |
|  | 23b | Discuss any limitations of the evidence included in the review. | Page 23-24 |
|  | 23c | Discuss any limitations of the review processes used. | Page 23-24 |
|  | 23d | Discuss implications of the results for practice, policy, and future research. | Page 22-24 |
| **OTHER INFORMATION** | | |  |
| Registration and protocol | 24a | Provide registration information for the review, including register name and registration number, or state that the review was not registered. | Page 2 |
|  | 24b | Indicate where the review protocol can be accessed, or state that a protocol was not prepared. | Page 2 |
|  | 24c | Describe and explain any amendments to information provided at registration or in the protocol. | Page 26 |
| Support | 25 | Describe sources of financial or non-financial support for the review, and the role of the funders or sponsors in the review. | Page 25 |
| Competing interests | 26 | Declare any competing interests of review authors. | Page 25 |
| Availability of data, code and other materials | 27 | Report which of the following are publicly available and where they can be found: template data collection forms; data extracted from included studies; data used for all analyses; analytic code; any other materials used in the review. | 25 |

*From:* Page MJ, McKenzie JE, Bossuyt PM, Boutron I, Hoffmann TC, Mulrow CD, et al. The PRISMA 2020 statement: an updated guideline for reporting systematic reviews. BMJ 2021;372:n71. doi: 10.1136/bmj.n71. This work is licensed under CC BY 4.0. To view a copy of this license, visit <https://creativecommons.org/licenses/by/4.0/>

**Supplementary File B**: MOOSE (Meta-analyses Of Observational Studies in Epidemiology) Checklist

A reporting checklist for Authors, Editors, and Reviewers of Meta-analyses of Observational Studies. You must report the page number in your manuscript where you consider each of the items listed in this checklist. If you have not included this information, either revise your manuscript accordingly before submitting or note N/A.

| **Reporting Criteria** | **Reported (Yes/No)** | **Reported on Page No.** |
| --- | --- | --- |
| **Reporting of Background** |  |  |
| Problem definition | Yes | 4 |
| Hypothesis statement | Yes | 5-6 |
| Description of Study Outcome(s) | Yes | 5-6  107-108 |
| Type of exposure or intervention used | Yes | 4 |
| Type of study design used | Yes | 1, 5-6 |
| Study population | Yes | 6 |
| **Reporting of Search Strategy** |  |  |
| Qualifications of searchers (eg, librarians  and investigators) | Yes | 8 |
| Search strategy, including time period  included in the synthesis and keywords | Yes | 8, Supplementary File C |
| Effort to include all available studies,  including contact with authors | Yes | 6-9 |
| Databases and registries searched | Yes | 8 |
| Search software used, name and  version, including special features used  (eg, explosion) | Yes | 8, Supplementary File C |
| Use of hand searching (eg, reference  lists of obtained articles) | No | N/A |
| List of citations located and those  excluded, including justification | No | Supplementary Table S1 |
| Method for addressing articles  published in languages other than  English | Yes | 6 |
| Method of handling abstracts and  unpublished studies | Yes | 8 |
| Description of any contact with authors | Yes | 10 |
| **Reporting of Methods** |  |  |
| Description of relevance or appropriateness of studies assembled for assessing the hypothesis to be tested | Yes | 4-6 |
| Rationale for the selection and coding of  data (eg, sound clinical principles or convenience) | No | N/A |
| Documentation of how data were  classified and coded (eg, multiple raters, blinding, and interrater reliability) | Yes | 8-9 |
| Assessment of confounding (eg,  comparability of cases and controls in studies where appropriate) | Yes | 6-11 |
| **Reporting Criteria** |  |  |
| Assessment of study quality, including  blinding of quality assessors; stratification or regression on possible predictors of study results | Yes | 8-11, Figure S1, Supplementary Table S2 |
| Assessment of heterogeneity | Yes | 11 |
| Description of statistical methods (eg,  complete description of fixed or random effects models, justification of whether the chosen models account for predictors of study results, dose-response models, or cumulative meta-analysis) in sufficient detail to be replicated | Yes | 9-11 |
| Provision of appropriate tables and  graphics | Yes | 12, 15-18, 20-21 |
| **Reporting of Results** |  |  |
| Table giving descriptive information for  each study included | Yes | 15-16 |
| Results of sensitivity testing (eg,  subgroup analysis) | No | 17-19 |
| Indication of statistical uncertainty of  findings | Yes | 19, Supplementary File E |
| **Reporting of Discussion** |  |  |
| Quantitative assessment of bias (eg,  publication bias) | Yes | 21-24 |
| Justification for exclusion (eg, exclusion  of non–English-language citations) | Yes | 13, Supplementary Table S3 |
| Assessment of quality of included studies | Yes | 21-24, Supplementary Figure S1, Supplementary Table S2 |
| **Reporting of Conclusions** |  |  |
| Consideration of alternative explanations  for observed results | Yes | 21-24 |
| Generalization of the conclusions (ie,  appropriate for the data presented and within the domain of the literature review) | Yes | 21-24 |
| Guidelines for future research | Yes | 21-24 |
| Disclosure of funding source | Yes | 25 |

**Supplementary File C**: Medline Search strategy

1. SARS-CoV-2/ or COVID-19/

2. ((corona* or corono*) adj1 (virus* or viral* or virinae*)).tw,kf.

3. (coronavirus* or coronovirus* or coronavirinae* or CoV or CoV2 or CoV-2 or HCoV*).tw,kf

4. (2019-novel OR 2019nCoV OR 2019-nCoV OR COVID-19 OR COVID19 OR COVID-2019 OR COVID2019 OR CONVID-19 OR CONVID19 OR CORVID-19 OR CORVID19 OR CoV2 OR CoV-2 OR HCoV* OR Ncov* OR Ncorona* OR Ncorono* OR NcovChina* OR NcovChinese* OR NcovHubei* OR NcovWuhan* OR SARS2 OR SARS-2 OR SARScoronavirus2 OR SARScoronavirus-2 OR SARScoronovirus2 OR SARScoronovirus-2 OR SARSCov19 OR SARSCov-19 OR SARS-CoV-2 OR SARSCoV-2 OR SARSCoV2 OR WN-CoV OR WNCoV OR wuhan-virus).tw,kf,ot.

5. ((pneumonia* OR outbreak* OR respiratory-illness* OR respiratory-disease* OR respiratory-symptom* OR seafood-market* OR food-market* OR wildlife) AND (Wuhan OR China OR Chinese OR Hubei OR Huanan)).tw,kf,ot.

6. ((new or novel or nouveau or 19 or 2019 or Wuhan or Hubei or Huanan or China or Chinese) adj3 (coronavirus* or corona virus* or betacoronavirus* or CoV or HCoV)).tw,kf,ot. 7. (longCOVID* or postCOVID* or postcoronavirus* or postSARS*).ti,ab,kf,ot.

8. (coronavirus/ or betacoronavirus/ or coronavirus infections/) and (disease outbreaks/ or epidemics/ or pandemics/)

9. ((coronavirus* or corona-virus* or betacoronavirus*) adj3 (pandemic* or epidemic* or outbreak* or crisis)).tw,kf,ot.

10. 1 or 2 or 3 or 4 or 5 or 6 or 7 or 8	or 9

11. exp emergency medical services/

12. exp emergency service, hospital/

13. (ED or emergency or "a&e").tw,kf,hw.

14. exp emergency medicine/

15. 11 or 12 or 13 or 14

16. exp Mental Disorders/

17. Mental Health/

18. exp Self-Injurious Behavior/

19. Child Psychiatry/

20. Adolescent Psychiatry/

21. psychology, adolescent/ or psychology, child/

22. (mental-health or mental-disorder* or suicid* or self-harm or self-injur* or non-suicid* or self-inflict* or self-wound* or parasuicid* or para-suicid* or self-poison* or self-mutilat* or self-cut* or self-scratch or self-burn or self-immolat* or suicide-attempt or self-batter* or NSSI or DSH or self-destruct* or (attempt* adj4 suicid*) or eating-disorder* or anorexi* or bulimi*).tw,kf.

23. 16 or 17 or 18 or 22

24. (kinder or kinders or kindergarten* or kinder-aged or boy or boys or girl or girls or child or children or childhood or pediatric* or paediatric* or school-age* or schoolage* or schoolchild* or schoolgirl* or schoolboy* or adolescen* or youth or youths or teen or teens or teenage* or ((young or emerging) adj (adult* or people or person*)) or AYA or AYAs).tw,kf.

25. 10 and 15 and 23 and 24

26. 10 and 15 and (19 or 20 or 21)

27. 25 or 26

28. limit 27 to english language

29. exp Australia/

30. (Sydney or Melbourne or Canberra or Brisbane or Perth or Adelaide or Darwin or Hobart).tw,kf.

31. (Australia* or Victoria or New South Wales or Queensland or Northern Territory or Tasmania).tw,kf.

32. 29 or 30 or 31

33. 28 and 32

**Supplementary File D**: Covidence data extraction instrument for systematic review

This file contains screenshots of the Covidence data extraction instrument used to collect information from included studies. This instrument was created by JND in Covidence, with feedback from SC, RB, KK, and GM.

Image 1. General information and study characteristics

Image 2. General information and study characteristics by exposure

Image 3. Demographics: outcomes and test statistics

Image 4. Diagnoses and clinical characteristics by exposure

Image 5. Diagnoses and clinical characteristics: outcomes and test statistics

**Supplementary File E**: Grading of Recommendations, Assessment, Development and Evaluation (GRADE) profile and summary of evidence table

This file contains a shortened GRADE profile table, providing a summary assessment of the certainty of evidence. This table was created by JND in GRADEpro.

| **COVID-19 compared to Pre-COVID-19 in examining changes in emergency department mental health use by children and young people** | | | | | | | |
| --- | --- | --- | --- | --- | --- | --- | --- |
| **Certainty assessment** | | | | | | | **Relative effect^†^ (95% CI)** |
| **Participants (studies) Follow-up** | **Risk of bias** | **Inconsistency** | **Indirectness** | **Imprecision** | **Other considerations^*^** | **Overall certainty of evidence** |  |
| **Overall psychiatric presentations** | | | | | | | |
| 79224 (4 non-randomised studies) | not serious^a^ | very serious^b^ | not serious^c^ | serious^d^ | None^e^ | ⨁◯◯◯ Very low^a,b,c,d^ | **Rate ratio 1.22** (1.00 to 1.49) |
| **Self-harm, suicide attempt, and suicidal ideation** | | | | | | | |
| 164866 (5 non-randomised studies) | serious^f^ | very serious^b^ | not serious^c^ | serious^g^ | none^e^ | ⨁◯◯◯ Very low^b,c,f,g^ | **Rate ratio 1.35** (1.00 to 1.82) |
| **Neurodevelopmental and behavioural disorder-related presentations** | | | | | | | |
| 7219 (3 non-randomised studies) | serious^h^ | very serious^b^ | not serious^c^ | serious^d^ | none^e^ | ⨁◯◯◯ Very low^b,c,d,h^ | **Rate ratio 1.12** (0.69 to 1.81) |
| **Eating disorder presentations** | | | | | | | |
| 2377 (3 non-randomised studies) | serious^h^ | serious^i^ | not serious^c^ | serious^d^ | strong association^j^ | ⨁◯◯◯ Very low^c,d,h,i^ | **Rate ratio 2.16** (1.72 to 2.72) |
| **Substance-related and addictive disorder presentations** | | | | | | | |
| 11331 (3 non-randomised studies) | serious^h^ | very serious^b^ | not serious^c^ | serious^d^ | none^e^ | ⨁◯◯◯ Very low^b,c,d,h^ | **Rate ratio 1.23** (0.86 to 1.75) |

**CI:** confidence interval
***Other considerations:** Publication bias, Large effect, Plausible confounding, Dose response relationship

**†Summary of findings:** Assumed risk not reported. Included studies used heterogeneous denominators for effect estimation, precluding direct comparison of absolute rates across studies. A rate ratio derived from standardised monthly presentation rates was used as the primary effect measure; see methods for details.

Explanations

a. All four included studies are retrospective and administrative in nature, and none were prospectively designed to examine this question. Variability in diagnostic coding practices is a potential concern; however, all four studies used statewide aggregated datasets processed by data custodians to standardise coding across hospitals, partially mitigating this risk. All four studies also had high weighted scores on the MASTER scale relative to the highest scoring observational study in this systematic review (range 91–96/100). As all included studies share the same retrospective observational design, relative scores reflect genuine methodological differences between studies rather than design class differences. On balance, risk of bias was not considered a serious concern for this outcome.

b. Inconsistency was considered to be high due to heterogeneity in how study populations, control and exposure periods, and outcomes were defined between studies. This is reflected by extremely high heterogeneity in meta-analysis of monthly rate-ratios (I² = 96.8-99.5%).

c. Population, intervention, comparator, and outcome were considered sufficiently direct overall. Minor concerns noted: age ranges varied across contributing studies; the COVID-19 period encompassed multiple concurrent factors beyond restrictions alone; and variability in diagnostic coding practices across hospitals introduced some uncertainty regarding outcome comparability. Indirectness was not considered a serious concern for any outcome.

d. Despite relatively high sample sizes within the included studies, the total number of included studies is low, so optimal information size is unlikely to be met. Therefore, the risk of imprecision is considered to be serious.

e. Publication bias could not be formally assessed as fewer than ten studies contributed to each meta-analysis, precluding reliable use of Egger's test or funnel plot asymmetry testing. No dose-response gradient or plausible confounding effect was identified.

f. All five included studies are retrospective and administrative in nature. Coding variability is a potential concern; however, three of five studies used statewide aggregated datasets, where coding is standardised during data aggregation by data custodians, partially mitigating this risk. The remaining two studies were conducted within single health networks. Four studies had high MASTER scale scores relative to the highest scoring observational study in this review (91–96/100); one scored lower (78/100), losing points for equal ascertainment, equal implementation, and sufficient analysis. This study was the only contributor not to identify an effect; whilst state-level variation may explain this, it cannot be confirmed. Risk of bias was considered a serious concern.

g. Downgraded one level for imprecision. Despite a large total sample (n = 164,500 across five studies), the pooled confidence interval (RR 1.35, 95% CI 1.00–1.82) includes unity, meaning a null effect cannot be excluded. The limited number of contributing studies was also considered in this judgement

h. All three included studies are retrospective and administrative in nature. Coding variability is a potential concern; however, two of three studies used statewide aggregated datasets, where coding is standardised during data aggregation by data custodians, partially mitigating this risk. The remaining study was conducted within a single health network. Two studies had high MASTER scale scores relative to the highest scoring observational study in this review (91/100); one scored lower (78/100), losing points for equal ascertainment, equal implementation, and sufficient analysis. Whilst state-level variation may explain some of the methodological differences between studies, this cannot be confirmed. Risk of bias was considered a serious concern.

i. Despite very high inconsistency (I² = 84.8%), the three contributing studies showed relatively consistent direction and magnitude. The I² value is meaningfully lower than those of other outcomes - however, it remains substantially higher than the generally accepted 75% threshold.

j. Publication bias could not be formally assessed due to insufficient contributing studies. A large effect was identified (pooled RR 2.16, 95% CI 1.72–2.72), with consistent increases across all three contributing studies, supporting upgrading of certainty by one level. No dose-response gradient or plausible confounding effect was identified.

**Figure S1**. Raw count of subscale scores on the MASTER scale for each study included in the systematic review

MASTER Scale subscale scores (Count)

|  | Format recruitment | Equal retention | Equal ascertainment | Equal implementation | Equal prognosis | Sufficient analysis | Temporal precedence |
| --- | --- | --- | --- | --- | --- | --- | --- |
| Carison 2022 | 2 | 2 | 3 | 4 | 2 | 3 | 2 |
| Cheek 2020 | 2 | 2 | 2 | 4 | 0 | 3 | 2 |
| Gatenby 2023 | 2 | 5 | 3 | 4 | 3 | 3 | 3 |
| Hiscock 2022 | 2 | 3 | 3 | 4 | 3 | 3 | 3 |
| Hu 2022 | 1 | 5 | 3 | 4 | 3 | 3 | 3 |
| Jones 2024 | 2 | 3 | 3 | 4 | 3 | 3 | 3 |
| Khan 2023 | 2 | 3 | 3 | 4 | 3 | 3 | 3 |
| Newberry-Dupe 2023 | 2 | 3 | 3 | 4 | 3 | 3 | 3 |
| Page 2021 | 2 | 5 | 3 | 4 | 3 | 3 | 3 |
| Sara 2023 | 2 | 4 | 3 | 4 | 3 | 3 | 3 |
| Sperandei 2022 | 2 | 2 | 3 | 4 | 3 | 3 | 2 |
| Sveticic 2021 | 2 | 3 | 3 | 4 | 3 | 3 | 3 |
| Tedja 2023 | 2 | 4 | 2 | 2 | 3 | 2 | 3 |

**Table S1**. Characteristics of studies excluded after full text review

|  |  | Location* | Setting | Study design | Age of Sample**  (years) | Visit types included | Reason excluded |
| --- | --- | --- | --- | --- | --- | --- | --- |
|  | Title |  |  |  |  |  |  |
| Dragovic 2020 | Emergency department mental health presentations before and during the COVID-19 outbreak in Western Australia | Perth, WA | Emergency department | Pre-post design | All ages | Mental health | Adult population: No disaggregation of age groups |
| Han 2022 | Rapid onset functional tic-like behaviours in children and adolescents during COVID-19: Clinical features, assessment and biopsychosocial treatment approach | Sydney, NSW | Tic Clinic | Interrupted time series | <18 | Rapid onset functional tic disorder | Wrong setting: Clinic not located in ED |
| Harris 2021 | Impact of COVID-19 social restrictions on trauma presentations in South Australia | SA | Emergency department | Pre-post design | All ages: disaggregated, 16-25 | Trauma presentations | Wrong patient population: Intentional self-injury not disaggregated by age |
| Horvat 2022 | Rising Paediatric Encounters for Mental Health Diagnoses Amidst Pandemic Mitigation Strategies in New South Wales | NSW | Emergency department | Commentary | 0-17 | Overall psychiatric | Wrong study design: Commentary on Hu 2021 |
| Skinner 2024 | Mental health impacts of COVID-19: A retrospective analysis of dynamic modelling projections for Australia | Australia | Emergency department | Interrupted time series using modelled data | 15-24 | Self-harm and suicide | Wrong study design: Modelled data |
| Springall 2022 | Impact of the coronavirus pandemic on anorexia nervosa and atypical anorexia nervosa presentations to an Australian tertiary paediatric hospital | Melbourne, VIC | Hospital eating disorder service | Interrupted time series | <18 | Anorexia Nervosa and Atypical Anorexia Nervosa | Wrong setting: Clinic not located in ED |
| Tao 2023 | Impacts of PM2.5 before and after COVID-19 outbreak on emergency mental disorders: A population-based quasi-experimental and case-crossover study | Hefei, China | Emergency Department | Interrupted time series | All ages | Organic and symptomatic mental disorders  Substance use disorders  Schizophrenia, schizotypal and delusional disorders  Mood disorders  Neurotic, stress-related, and somatoform disorders | Wrong location: China |

*Locations abbreviated: NSW = New South Wales; VIC = Victoria; QLD = Queensland; WA = Western Australia; SA = South Australia

**Age of study participants <25 years. Excludes study participants aged >24 years.

**Table. S2.** Raw count, summary statistics, and weighted scores for subscales and total instrument on the MASTER scale for each study included in the systematic review

|  | Format recruitment | Equal retention | Equal ascertainment | Equal implementation | Equal prognosis | Sufficient analysis | Temporal precedence | Total | Weighted score |
| --- | --- | --- | --- | --- | --- | --- | --- | --- | --- |
| Carison 2022 | 2 | 2 | 3 | 4 | 2 | 3 | 2 | 18 | 78 |
| Cheek 2020 | 2 | 2 | 2 | 4 | 0 | 3 | 2 | 15 | 65 |
| Gatenby 2023 | 2 | 5 | 3 | 4 | 3 | 3 | 3 | 23 | 100 |
| Hiscock 2022 | 2 | 3 | 3 | 4 | 3 | 3 | 3 | 21 | 91 |
| Hu 2022 | 1 | 5 | 3 | 4 | 3 | 3 | 3 | 22 | 96 |
| Jones 2024 | 2 | 3 | 3 | 4 | 3 | 3 | 3 | 21 | 91 |
| Khan 2023 | 2 | 3 | 3 | 4 | 3 | 3 | 3 | 21 | 91 |
| Newberry-Dupe 2023 | 2 | 3 | 3 | 4 | 3 | 3 | 3 | 21 | 91 |
| Page 2021 | 2 | 5 | 3 | 4 | 3 | 3 | 3 | 23 | 100 |
| Sara 2023 | 2 | 4 | 3 | 4 | 3 | 3 | 3 | 22 | 96 |
| Sperandei 2022 | 2 | 2 | 3 | 4 | 3 | 3 | 2 | 19 | 83 |
| Sveticic 2021 | 2 | 3 | 3 | 4 | 3 | 3 | 3 | 21 | 91 |
| Tedja 2023 | 2 | 4 | 2 | 2 | 3 | 2 | 3 | 18 | 78 |
| Total | 25 | 44 | 37 | 50 | 35 | 38 | 36 |  |  |
| Total possible score | 4 | 5 | 7 | 6 | 6 | 3 | 5 | 36 |  |
| Total possible all studies | 52 | 65 | 91 | 78 | 78 | 39 | 65 |  |  |
| Average proportion of safeguard met (%) | 48% | 68% | 41% | 64% | 45% | 97% | 55% |  |  |
|  |  |  |  |  |  |  |  |  |  |
| median | 2 | 3 | 3 | 4 | 3 | 3 | 3 | 21 | 91 |
| Upper limit | 2 | 5 | 3 | 4 | 3 | 3 | 3 | 23 | 96 |
| Lower limit | 1 | 2 | 2 | 2 | 0 | 2 | 2 | 15 | 83 |

**Table. S3**. Studies included and excluded from the meta-analysis

| ***Inclusion/exclusion*** Study/year | **State** | **Reason for inclusion/exclusion** |
| --- | --- | --- |
| **Overall psychiatric** | | |
| ***Included*** | | |
| Hu 2022 | NSW | Only NSW study looking at overall psychiatric |
| Jones 2024 | QLD | Largest QLD study looking at overall psychiatric (State-wide) |
| Hiscock 2022 | VIC | Largest VIC study looking at overall psychiatric (State-wide, ages 0-17) |
| Newberry-Dupe 2023 | VIC | Largest VIC study looking at overall psychiatric (State-wide, ages 18-24) |
| ***Excluded*** | | |
| Khan 2023 | NSW, QLD, VIC, SA, WA | Contains data captured in above studies & not disaggregated by region |
| Tedja 2023 | QLD | Single health network, data captured in Jones 2024 |
| Carison 2022 | VIC | Single site, data captured in Hiscock 2022 |
| Cheek 2020 | VIC | Two health networks & short control / exposure timelines, data captured in Hiscock 2022 |
| **Self-harm, suicidal ideation, and suicide attempt** | | |
| ***Included*** | | |
| Sara 2023 | NSW | Largest NSW study looking at self-harm (State-wide) |
| Sveticic 2021 | QLD | QLD study looking at self-harm (Gold Coast) |
| Tedja 2023 | QLD | QLD study looking at self-harm (Brisbane) |
| Hiscock 2022 | VIC | Largest VIC study looking at self-harm (State-wide, ages 0-17) |
| Newberry-Dupe 2023 | VIC | Largest VIC study looking at self-harm (State-wide, ages 18-24) |
| ***Excluded*** | | |
| Khan 2023 | NSW, QLD, VIC, SA, WA | Contains data captured in above studies & not disaggregated by region |
| Gatenby 2023 | NSW | Single health network, data captured in Sara 2023 |
| Page 2021 | NSW | Single site, data captured in Sara 2023 |
| Sperandi 2022 | NSW | Single health network, data captured in Sara 2023 |
| Carison 2022 | VIC | Single site, data captured in Hiscock 2022 |
| **Neurodevelopmental and behavioural disorders** | | |
| ***Included*** | | |
| Tedja 2023 | QLD | Only QLD study looking at neurodevelopmental/behavioural disorders |
| Hiscock 2022 | VIC | Largest VIC study looking at neurodevelopmental/behavioural disorders (State-wide, ages 0-17) |
| Newberry-Dupe 2023 | VIC | Largest VIC study looking at neurodevelopmental/behavioural disorders (State-wide, ages 18-24) |
| ***Excluded*** | | |
| Khan 2023 | NSW, QLD, VIC, SA, WA | Contains data captured in above studies & not disaggregated by region |
| Carison 2022 | VIC | Single site, data captured in Hiscock 2022 |
| **Eating disorders** | | |
| ***Included*** | | |
| Tedja 2023 | QLD | Only QLD study with available data on eating disorders |
| Hiscock 2022 | VIC | Largest VIC study looking at eating disorders (State-wide, ages 0-17) |
| Newberry-Dupe 2023 | VIC | Largest VIC study looking at eating disorders (State-wide, ages 18-24) |
| ***Excluded*** | | |
| Khan 2023 | NSW, QLD, VIC, SA, WA | Contains data captured in above studies & not disaggregated by region |
| Carison 2022 | VIC | Single site, data captured in Hiscock 2022 |
| **Substance related and addictive disorders** | | |
| ***Included*** | | |
| Tedja 2023 | QLD | Only QLD study looking at substance use disorders |
| Hiscock 2022 | VIC | Largest VIC study looking at substance use disorders (State-wide, ages 0-17) |
| Newberry-Dupe 2023 | VIC | Largest VIC study looking at substance use disorders (State-wide, ages 18-24) |
| ***Excluded*** | | |
| Khan 2023 | NSW, QLD, VIC, SA, WA | Contains data captured in above studies & not disaggregated by region |
